# Novel single base-pair deletion in exon 1 of *XK* gene leading to McLeod syndrome with chorea, muscle wasting, peripheral neuropathy, acanthocytosis and haemolysis

**DOI:** 10.1016/j.jns.2014.01.034

**Published:** 2014-04-15

**Authors:** Sarah Wiethoff, Georgia Xiromerisiou, Conceição Bettencourt, Anna Kioumi, Iakovos Tsiptsios, Athanasios Tychalas, Markousi Evaggelia, Kaltsounis George, Vasileios Makris, John Hardy, Henry Houlden

**Affiliations:** a Department of Molecular Neuroscience, UCL Institute of Neurology and National Hospital for Neurology and Neurosurgery, London, UK; b MRC Centre for Neuromuscular Diseases, UCL Institute of Neurology and National Hospital for Neurology and Neurosurgery, London, UK; c Department of Neurology, Papageorgiou Hospital, Thessaloniki, Greece; d Department of Haematology, Papageorgiou Hospital, Thessaloniki, Greece; e Orthopedic Department, Papageorgiou Hospital, Thessaloniki, Greece

**Keywords:** McLeod syndromes, XK gene, Novel mutation, Frameshift deletion, Acanthocytosis, Chorea, Non-CGD

## Abstract

We present a 70-year-old male patient of Greek origin with choreatic movements of the tongue and face, lower limb muscle weakness, peripheral neuropathy, elevated creatinephosphokinase (CPK), acanthocytosis and haemolysis in the absence of Kell RBC antigens with an additional Factor IX-deficiency. Genetic testing for mutations in the three exons of the *XK* gene revealed a previously unreported hemizygous single base-pair frameshift deletion at exon 1 (c.229delC, p.Leu80fs). In conclusion, we hereby describe a rare phenotype of a patient with McLeod syndrome which was discovered coincidentally during routine blood group testing and consecutively genetically confirmed.

## Highlights

• McLeod syndrome with chorea, muscle wasting, and peripheral neuropathy
• Acanthocytosis and haemolysis in the absence of Kell RBC antigens
• McLeod syndrome with an additional Factor IX deficiency
• Novel hemizygous single base-pair frameshift deletion in the *XK* gene

## 1 Introduction

McLeod syndrome is a rare X-linked multisystemic disease caused by mutations in the *XK* gene, which is thought to encode for a membrane transporter [1]. This rare syndrome together with autosomal recessive chorea-acanthocytosis forms the core of the neuroacanthocytosis syndromes, both featuring an estimated prevalence of less than 1–5/1,000,000 [2]. With an onset of neurological symptoms mostly around the 4th decade, the McLeod syndrome is a slowly progressive multisystemic disease comprising acanthocytosis, haemolysis, chronic anaemia, hepatosplenomegaly, cardiomyopathy, myopathy, neuropathy, choreatic movement disorders, psychiatric manifestations and cognitive decline as the most common symptoms, with additional occurrence of more rare multi-system features being possible [3]. Not only the clinical phenotype of McLeod syndrome but also the mutational spectrum of the *XK* gene shows heterogeneity, with gross deletions, spanning the entire or a significant part of the *XK* coding region, as well as small frameshift indels, and single nucleotide substitutions described as associated with the phenotype. Their common ground is the prediction of an absent or truncated, non-functional gene product [3]. We hereby present a patient with a novel *XK* frameshift mutation associated with a McLeod syndrome without signs of chronic granulomatous disease (CGD).

## 2 Case report

A 70-year-old Greek man was referred to the Neurology Department of the Papageorgiou Hospital in Thessaloniki. The main initial finding was choreatiform tongue movements reported by the haematology unit that detected a weak expression of K red blood cell antigens, acanthocytosis, haemolysis, and a form of Factor IX deficiency when screening his blood type prior to a blood transfusion needed for a scheduled surgical procedure. Those tongue movements were noted recently and were attributed by the family and the proband to his ill-fitting denture. The patient also had a slight generalised restlessness with frequent changes of posture but without any tic-like or choreiform movements of any particular part of his body apart from his tongue and the face. Neurological findings on examination included chorea of the face and tongue, and lower limb muscle weakness with muscle wasting.

His past medical history includes severe progressive skeletal deformities after the age of 50, with osteoarthritis of the knees bilaterally that resulted in severe varus deformities of the knees.

**Fig. 1:**
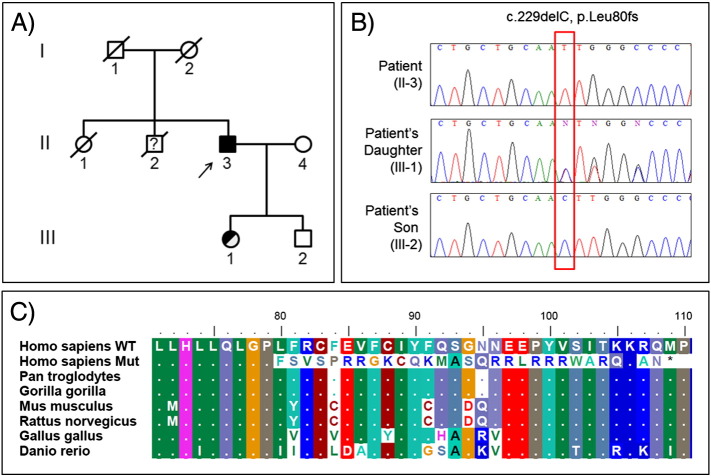
A) Family pedigree. The arrow indicates the proband. Black filled symbols indicate affected individuals, while half-filled indicate unaffected carriers of the mutation; B) Chromatograms depicting the mutation segregating in this family; C) Protein alignment showing conserved positions across species and the very premature stop codon (marked with the asterisk) caused by the c.229delC frameshift mutation (WT — human wild-type allele; Mut — human mutant allele).

The patient had a brother who died at the age of 60, and presented with severe facial dyskinesias, chorea and schizophrenia like psychosis. His sister, who died at the age of 70 after an acute ischemic stroke, was referred as being healthy until that incident. His 38-year-old daughter and his 35-year-old son are both healthy (Fig. 1A). There is no additional family history regarding neuropsychiatric diseases.

## 3 Clinical investigations

**Fig. 2:**
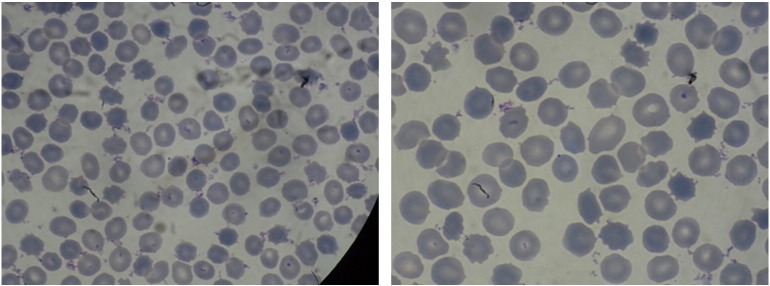
Acanthocytosis in peripheral blood. The smear shows frequent acanthocytes, representing ~ 19% of all red blood cells.

**Fig. 3:**
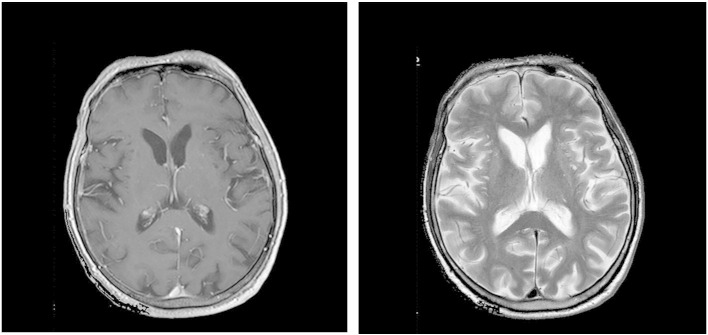
Proband's T1- (left panel) and T2-weighted axial (right panel) MRI at time of presentation without major abnormalities.

Laboratory investigation revealed blood type group A1, DAT negative (−), IAT positive (+). Antibody identification panel testing demonstrated panagglutination of the same intensity in all cellular lines in the LISS/Coombs phase. Enzyme phase, 4 °C incubation phase and auto control (A/C) were negative. His detailed blood group profile was: C (+), c (+), D (+), E (−), e (+), Cw (−), K (−), k (−), Kpa (−), Kpb (−), P1 (+), Lea (−), Leb (+), Lua (−), Lub (+), Jka (+), Jkb (+), M (+), N (+), S (+), s (+), Fya (−), and Fyb (+). Peripheral blood smear revealed the existence of acanthocytes (Fig. 2) at a percentage of 19% of all red blood cells. Other abnormal laboratory values included: Factor IX 8% (normal values 60−150%), Activated Partial Thromboplastin Time (APTT) 58.1 (normal value 30.0 s), Partial Thromboplastin Time Lupus Anticoagulans (PTT-LA) 61.3 (normal value < 45 s), Lactate dehydrogenase (LDH) 299 U/l (normal values 135–225), creatinephosphokinase 1400 U/l (normal range in males 24–195 U/l). His further routine blood tests were normal. Electrophysiological examination showed diffuse chronic sensorimotor peripheral neuropathy of axonal type. A brain MRI did not show any abnormal findings apart from mild cortical atrophy (Fig. 3). Neuropsychological investigation and EEG were normal. The patient did not present any cardiac complications and had a normal echocardiogram and 24-hour monitoring.

## 4 Genetic analysis

Sequence analysis of the three exons of the *XK* gene detected a hemizygous single base-pair frameshift deletion (Fig. 1B) at exon 1 (c.229delC, p.Leu80fs), leading to a very premature stop codon (Fig. 1C). Exons 2 and 3 revealed no further mutations in this gene. Primers and conditions are available upon request. The deletion is absent in the patient's healthy son and is present in his unaffected daughter in the heterozygous state (Fig. 1A–B). To the best of our knowledge, this variant has not been previously reported and is not present in public databases, such as dbSNP and exome variant server.

## 5 Discussion

McLeod syndrome belongs to the rare neuroacanthocytosis syndromes with approximately a few hundred cases worldwide [4]. The disease distribution lacks obvious clusters and McLeod syndrome has been described across Northern and Southern America, Europe, Japan and recently China [4,5]. It is heterogeneous in its clinical spectrum even within families (e.g., [6,7]), and its mutational spectrum is also wide. So far, gross deletions, including large parts of the *XK* gene or even nearby genes, small frameshift indels as well as point mutations have been described, all resulting in the absence or the truncation of a functional gene product [3].

In our patient the neurological phenotype became obvious rather late in life and his haematological abnormalities were revealed coincidentally during routine blood group testing. Interestingly, our patient exhibits a Factor IX-deficiency, which to the best of our knowledge has not yet been reported as co-occurring with McLeod syndrome.

The diagnosis of McLeod syndrome is generally based on the haematological findings of weak Kell antigens. Therefore, carriers of this phenotype could be diagnosed mainly in blood banks [5]. However, the majority of cases have been diagnosed by neurologists due to prominent neurological manifestations. The wide phenotypic spectrum of the disease and the insidious onset usually delays the diagnosis. Many patients could have been diagnosed properly if there was a follow up of all carriers of the McLeod blood group phenotype that are discovered coincidentally, as in the patient described here. The genetic testing in our patient revealed a novel single base-pair frameshift deletion at exon 1 of the *XK* gene, which would lead to a very premature stop codon. Even if the protein is produced it would lack more than 330 amino acids (normal size 444 amino acids). We hereby were able to expand the mutational spectrum of *XK* mutations leading to McLeod syndrome.

## Conflict of interest

The authors declare no conflict of interest.
